# Diet Quality Trajectories over Adulthood in a Biracial Urban Sample from the Healthy Aging in Neighborhoods of Diversity across the Life Span Study

**DOI:** 10.3390/nu15143099

**Published:** 2023-07-11

**Authors:** Marie Fanelli Kuczmarski, May A. Beydoun, Michael F. Georgescu, Nicole Noren Hooten, Nicolle A. Mode, Michele K. Evans, Alan B. Zonderman

**Affiliations:** Laboratory of Epidemiology and Population Sciences, National Institute on Aging, NIH, Baltimore, MD 21224, USA; baydounm@mail.nih.gov (M.A.B.); michael.geougescu@nih.gov (M.F.G.); norenhootenn@mail.nih.gov (N.N.H.); nicolle.mode@nih.gov (N.A.M.); evansm@grc.nia.nih.gov (M.K.E.); zondermana@gmail.com (A.B.Z.)

**Keywords:** diet quality, Healthy Eating Index, Dietary Inflammatory Index, group-based trajectory model, African American

## Abstract

Limited investigation has been done on diet quality trajectories over adulthood. The main study objectives were to determine the diet quality group trajectories (GTs) over time and to detect changes in a socio-economically and racially diverse middle-aged cohort. Data from three waves of the Healthy Aging in Neighborhoods of Diversity across the Life Span (HANDLS) study were used to determine diet quality with group-based trajectory modeling (GBTM). Three quality indices—the Healthy Eating Index (HEI), the Dietary Inflammatory Index (DII), and the Mean Adequacy Ratio (MAR)—were explored. The rate of change in quality over time was determined by mixed-effects regression analysis. Three diet quality GTs, low, middle, and high quality, were identified for each index and confirmed with spaghetti plots. Within each GT, only small changes in diet quality scores were observed, with improvements for the HEI and DII indices and a slight decline in MAR scores. Weighted kappa values revealed that the DII had better agreement with the HEI-2010 and MAR indices compared with the agreement between the HEI-2010 and MAR. Bayesian estimates revealed that the annualized rate of change in diet quality per person across the GTs was similar. There was minimal change in diet quality over time, regardless of the diet quality index used.

## 1. Introduction

Diet is a modifiable risk factor for several chronic conditions [1,2,3,4]. Throughout life, the quality of an individual’s dietary patterns can affect health in later years as well as life expectancy [5,6,7,8,9]. There is also evidence that dietary patterns during selected life stages can influence eating practices later in life [10,11,12,13]. For instance, food preferences early in life set the foundation for food choices and eating habits in later childhood and adulthood [14,15,16]. Findings from the Saskatchewan Pediatric Bone Mineral Accrual Study suggest that healthy dietary habits established during childhood and adolescence moderately continue into adulthood [17]. Data from the Baltimore Longitudinal Study of Aging, a study in the United States, revealed that improving diet quality, as assessed by the Alternate Healthy Eating Index-2010, in middle age could contribute to better physical function at older ages [18]. Middle-aged women enrolled in the Australian Longitudinal Study on Women’s Health whose diets were in the highest quality tertile initially and that improved in quality over 9 years gained significantly less weight compared with women whose diets were rated in the lower tertiles and that worsened in quality over time [19]. A better understanding of the quality of dietary patterns over time in adulthood may be valuable when targeting interventions to reduce the risk of the development of chronic conditions.

The group-based trajectory modeling (GBTM) method has been used to explore dietary pattern trajectories since 2014 [13,14,18,20]. This method uses longitudinal data to account for between-individual variation and describe the continuity of different behaviors of groups of individuals through time [21]. One study examining dietary pattern trajectories in Chinese adults found that the individuals in the trajectory with the greatest adherence to a meat dietary pattern were at greatest risk of overweight and obesity [22]. There are only a few studies using latent class methods to examine changes or stability in diet quality trajectories [13,14]. Mertens et al. reported that diet quality, measured by the Healthy Eating Index (HEI)-2010, changed in Flemish adults over 10 years [23]. Knowledge of groups of individuals with suboptimal diet quality and their demographic and environmental factors may assist health professionals when developing strategies for targeted interventions to close the gap between life expectancy and healthy life expectancy.

Dietary quality can be measured by different assessment tools. Although diet quality indices vary in scoring, all provide an overall picture of how a dietary pattern aligns with recommended food and/or nutrient intake guidance [24]. These indices can be used to measure changes in quality in people with and without chronic conditions. The HEI-2010 is based on the adequacy of food groups along with moderation of refined grains, sodium, and nutrients providing empty calories. The HEI has been shown to effectively measure changes in diet quality [25,26]. In contrast, both the Dietary Inflammatory Index (DII) and Mean Adequacy Ratio (MAR) include micronutrients, but their intakes are related to different end-points, namely the risk of effect on inflammatory markers [27] or the actual micronutrient Recommended Dietary Allowances of the individual [28], respectively. The DII has been used to assess changes in dietary quality over time [29,30,31], but no studies using the MAR were found in the current literature.

When exploring the associations of diet with disease, changes solely in dietary behaviors can fail to detect relationships or attenuations of observed effects [8]. Although important, evaluating the stability of diet quality in adulthood is rarely done [8,13]. The primary objectives of this study were to identify diet quality trajectories over time with GBTM and to assess the rate of change in these quality trajectories in African American and White socio-economically diverse participants from the Healthy Aging in Neighborhoods of Diversity across the Life Span (HANDLS) study. Three diet quality indices, namely the Healthy Eating Index, the Dietary Inflammatory Index, and the Mean Adequacy Ratio, were examined. The secondary objective was to compare sample characteristics across group trajectories using these indices.

## 2. Methods

### 2.1. HANDLS Study

The HANDLS study design has been described in detail elsewhere [32,33]. Briefly, HANDLS is a population-based cohort study designed to determine the role of race and socioeconomic status in health disparities observed between African American and White men and women. Participants were recruited as an area probability sample residing in 13 pre-determined neighborhoods in Baltimore City, MD, USA. The baseline cohort included 3720 community-dwelling individuals aged 30–64 years. 

### 2.2. Study Participants

**Figure 1:**
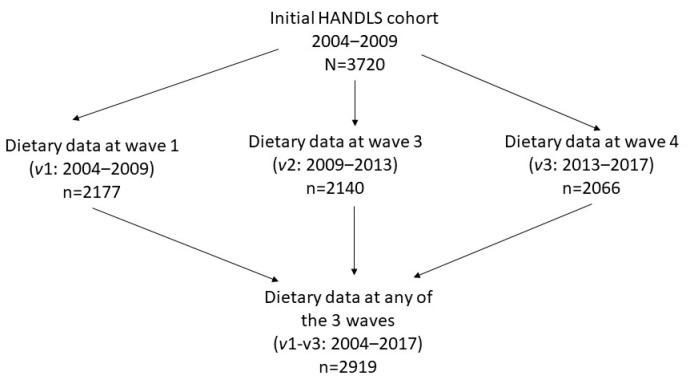
Flow diagram for study participation selection from the HANDLS study.

The individuals in this study represent adults interviewed and examined in Waves 1, 3, and 4 of the HANDLS study (n = 2919) (Figure 1). Wave 1 was the baseline wave initiated in August 2004 and completed in March 2009. Wave 3 was the first in-person follow-up wave and conducted between June 2009 and July 2013. Wave 4 was the second in-person follow-up wave conducted between September 2013 and September 2017. In this article, Wave 1 will be referred to as visit (*v*) 1, Wave 3 as *v* 2, and Wave 4 as *v* 3. Each participant provided written informed consent and was compensated monetarily. The study was conducted according to the guidelines of the Declaration of Helsinki, and the study protocol was approved by Human Institutional Review Board at the National Institutes of Health. 

### 2.3. Participant Characteristics

Race was self-reported as African American or White, and sex at birth was coded as male or female. Participants were categorized as above or below poverty status defined by 125% of the 2004 U.S. Health and Human Services Poverty Guidelines at baseline enrollment [34]. Educational attainment was coded as less than high school, high school, or more than high school. 

### 2.4. Dietary Collection Method

This study implemented the same dietary assessment method at each visit. Dietary data in the HANDLS study were collected by trained interviewers using the USDA Automated Multiple-Pass Method (AMPM) for the 24 h recall [35]. All foods and beverages reported were assigned USDA food codes using the USDA Food and Nutrient Database for Dietary Studies (FNDDS). For *v* 1 of the HANDLS study, foods and beverages were coded using FNDDS 3.0 (2005–2006), for *v* 2, FNDDS 5.0 (2009–2010), and for *v* 3, FNDDS 2013–2014 [36]. Both recalls in *v* 1 were collected in person. For *v* 2 and *v* 3, the first recall was obtained in person and the second by phone. Only those participants who completed two 24 hr dietary recalls were included in this study.

### 2.5. Diet Quality Indices

#### 2.5.1. Healthy Eating Index (HEI)-2010

The HEI-2010 is an index that measures compliance with the *Dietary Guidelines for Americans* [37]. A detailed description of the procedure used to calculate the HEI-2010 is available on the HANDLS website [38]. The National Cancer Institute’s Applied Research website provided the basic steps to calculate the HEI-2010 component and total scores and the statistical codes for 24 h dietary recalls [39]. For each visit, component and total HEI-2010 scores were calculated for each recall day and averaged to obtain the mean for both days combined. The maximum possible score was 100. 

#### 2.5.2. Dietary Inflammatory Index (DII)

The inflammatory potential of the diet was calculated using 35 parameters. The parameters included energy, alcohol, protein, carbohydrate, dietary fiber, total fat, saturated fat, monounsaturated fat, polyunsaturated fat, omega 3 fatty acids, omega 6 fatty acids, cholesterol, 11 vitamins, 4 minerals, 6 flavonoid classes, caffeine, and tea. The original calculation by Shivappa et al. also included the following 10 parameters: trans fatty acids, garlic, ginger, onion, pepper, rosemary, saffron, thyme/oregano, turmeric, and eugenol [27]. These were excluded because they were not included in the USDA FNDDS. Shivappa et al. reported that dropping from the maximum 45 to 28 parameters does not impair the DII predictive capability [40]. Their explanation that the omission of spices and trans fatty acids would not make a major impact on the DII score was due to their infrequency of consumption, and when consumed, the quantities of the spices and trans fats were small in the US. Applying the global composite database to data from the HANDLS study, the possible maximal pro-inflammatory DII score was +10.44 and the maximal anti-inflammatory DII score was −10.44. Typically, the higher the DII score, the more pro-inflammatory the dietary pattern.

#### 2.5.3. Mean Adequacy Ratio (MAR)

Nutrient-based diet quality was assessed using the MAR, which is calculated from Nutrient Adequacy Ratios [41]. Nutrient Adequacy Ratio is defined as the ratio of the participant’s daily intake of a nutrient to his/her current Recommended Dietary Allowance (RDA) for that nutrient [42]. The RDA was matched for the age and sex of participants, and vitamin C was adjusted for smokers [43,44]. Scores for 17 micronutrients contributed only by food: Vitamins A, C, D, E, B6, B12, folate, iron, thiamin, riboflavin, niacin, copper, zinc, calcium, magnesium, phosphorus, and selenium were calculated. These scores were then converted into a percent, with values exceeding 100 truncated to 100. The MAR formula was: MAR = (∑NAR scores)/17, with 100 as the maximal possible score [41]. All ratios for each visit were calculated for each recall day and then averaged.

### 2.6. Statistical Analyses

Multiple imputations (5 imputations, 10 iterations) were conducted using chained equations for the non-exposure and non-outcome variables with missing data. Using data from all 3 visits, the *traj* and *trajplot* Stata plugins for estimating GBTM were used to create the diet quality trajectories [45,46]. The plugin is adapted from a well-established SAS procedure [45], which identifies groups of individuals with similar developmental trajectories over time. This group-based approach utilizes a multinomial modeling strategy and maximum likelihood to estimate model parameters, with maximization achieved by the quasi-Newton procedure. We specified a censored normal distribution for the selected outcomes, with intercept (0), linear (1), quadratic (2), and cubic (3) orders for each group trajectory, and displayed group-based trajectories over time with 95% confidence intervals (CI). For consistency and ease of interpretation, we defined up to three groups per outcome. Adding more groups was also attempted, though estimated prevalence for these additional groups was <5% in some of the diet quality indices. For this reason, only 3 groups were chosen. We reviewed the Bayesian Information Criterion (BIC) for each GBTM model as a goodness-of-fit measure. Alternative models were compared per diet quality index using a difference in BIC of 20 points as a cutoff to decide on the most parsimonious model possible. The linear model was chosen when δBIC was smaller compared with the quadratic model by a number >20. This procedure was applied to all 3 diet quality indices. Age was used as the time variable in these models. Consequently, each index yielded trajectories for 3 groups. The coefficients with SE and p-values for each group derived from the group-based trajectory modeling were calculated. To determine overall agreement between diet trajectory groups for the three diet quality indices, cross-tabulations were conducted and a weighted kappa estimate was derived. In this method, more weight is placed on agreements between two similar groups, and the most weight is placed on exact matches between the groups.

The mixed-effects regression models (mixed command in Stata) were run using the diet trajectory groups, adjusting for age (centered at visit 1), sex, race, and poverty status. All models incorporated number of years elapsed between visits (variable *TIME*) with two-way interaction terms between sociodemographic covariates and *TIME*. Additional models assessing stability included diet quality trajectories among covariates, which also interacted with *TIME*. A reduced model with only linear *TIME* as the predictor for each diet quality index was used in order to obtain an empirical Bayes estimator for annualized rate of change per individual for each diet quality index. Square and cubic terms were included to allow the comparison of BIC using a similar criterion as for GBTM. Models were run for the overall sample, by sex, and by race. Spaghetti plots were presented using a 10% simple random sample of the participants in the final analytic sample (See Supplementary Figures S1–S3). These plots were performed to show the shape of each trajectory, whether linear or non-linear, and determine the level of stability over time. Observed values for the diet quality measures were plotted in a line for each participant by year since enrollment using R version 4.2 [47]. Those observed participant trajectories were plotted for each GBTM group to visualize both their initial level and rate of change over time, where available, without making model-based assumptions, including random missingness.

Multinomial logistic regression models were also conducted for each diet quality index in order to examine the association between the GBTM trajectory as a multi-level outcome and the baseline socio-demographic characteristics, mainly age, sex, race, and poverty status entered simultaneously, while considering the “low diet quality” category as the common referent. 

Stata and R scripts used in this manuscript are provided in github.com/baydounm/HANDLS_DieTTRAJPAPER.

## 3. Results

The mean ages of the HANDLS study participants included in this study were 48.4 ± 0.2 y at *v* 1 (n = 2919), 53.0 ± 0.2 y at *v* 2 (n = 2377), and 56.6 ± 0.2 y at *v* 3 (n = 2156). Over half the sample were women, African American adults, and had completed high school or more education. Approximately 40% of the overall sample was below the poverty line. The percentage was higher for women compared with men and for African Americans compared with White adults (Table 1).

The mean (±SE) scores of the HEI diet quality index at visits 1, 2, and 3 were 42.6 ± 0.3, 46.3 ± 0.3, and 48.7 ± 0.3, respectively. The mean HEI-2010 scores ranged from 42–49 out of a maximum of 100 at each visit, indicating only generally poor adherence to the *Dietary Guidelines for Americans*. The mean DII scores were positive, indicating a pro-inflammatory diet at each visit. At *v* 1, the DII was 3.29 ± 0.04; at *v* 2, it was 2.92 ± 0.04; and at *v* 3, it was 2.56 ± 0.05. The mean MAR scores at each visit were as follows: 77.1 ± 0.3 at *v* 1, 77.2 ± 0.3 at *v* 2, and 76.4 ± 0.3 at *v* 3. They were >67 but <80 out of a maximum of 100, suggesting micronutrient intakes were most likely adequate.

The GBTM analyses revealed three trajectory groups for each of the diet quality indices explored (Table S1). These three groups were labeled low, medium, and high diet quality. These groups were confirmed with spaghetti plots, which show the increasing/decreasing level of diet quality at each age and thus any intercept differences between the three GBTM groups. (Figures S1–S3). For the HEI and DII indices, there were improvements in diet quality trajectories, ranging from a positive 4.28 to 6.75 points in the HEI-2010 score and from a negative 0.20 to 1.99 points in the DII index. However, the MAR scores appear to decline minimally with time, from 1 to 2.4 points over the group trajectories. 

The mean (±SE) diet quality scores by trajectory for each visit are provided in Table 2. The Bayesian estimates of the mean diet quality index and slope across visits were based on observations derived from repeated measures on 2919 participants for the HEI-2010 and MAR and 2918 participants for the DII. The mean (±SE) for the HEI-2010 was 42.88 ± 0.07, with a slope of 0.66, suggesting a slight increase in score. The mean (±SE) DII was 3.29 ± 0.01, with a slope of −0.08, indicating a change toward anti-inflammatory potential. Lastly, the mean (±SE) MAR was 77.21 ± 0.08, with a slope of −0.08, suggesting a slight decline in scores. 

**Figure 2:**
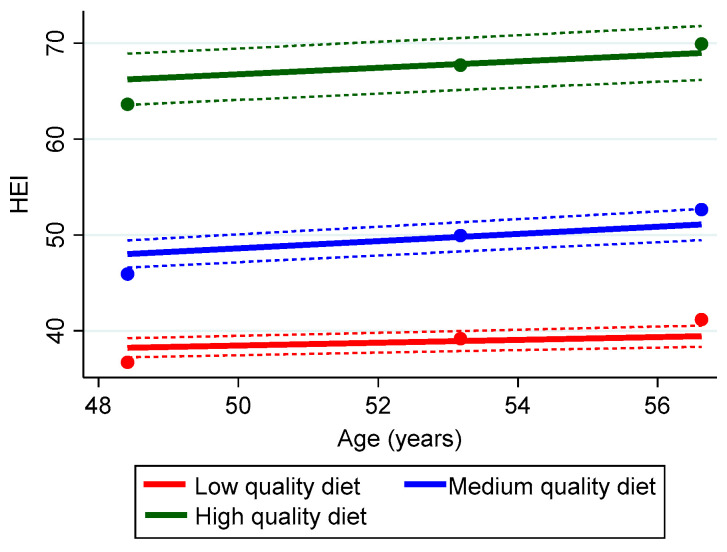
Group Trajectories of Diet Quality using the Healthy Eating Index (HEI)-2010 for the HANDLS study sample, 2004–2017 (n = 2919). Dots represent observed group means, solid lines represent the estimated trajectories, and dashed lines represent error estimates. Percentage of sample/group trajectory: Low, 46.1%; Medium, 48.3%; High, 5.6%.

As shown in Figure 2, the low diet quality trajectory, represented in red, was comprised of approximately 46.1% of the HANDLS study participants who had the lowest adherence to the HEI-2010. The mean (SD) Bayesian estimator of the annualized rate of change was 0.69 (0.08) (95% CI [0.53, 0.85]), suggesting slight improvement over time. An estimated 48.3% of the sample had HEI-2010 adherence scores higher than the previous group, as represented by the middle trajectory line in blue. The mean (SD) Bayesian estimator for the middle trajectory was 0.62 (0.06) (95% CI [0.49, 0.75]), indicating slight improvement over time. Only about 5.6% of the sample had a trajectory indicating a high HEI-2010 adherence score, represented by the green color. Their diet quality scores also improved over time: mean (SD) Bayesian estimator 0.80 (0.09) (95% CI [0.63, 0.97]).

**Figure 3:**
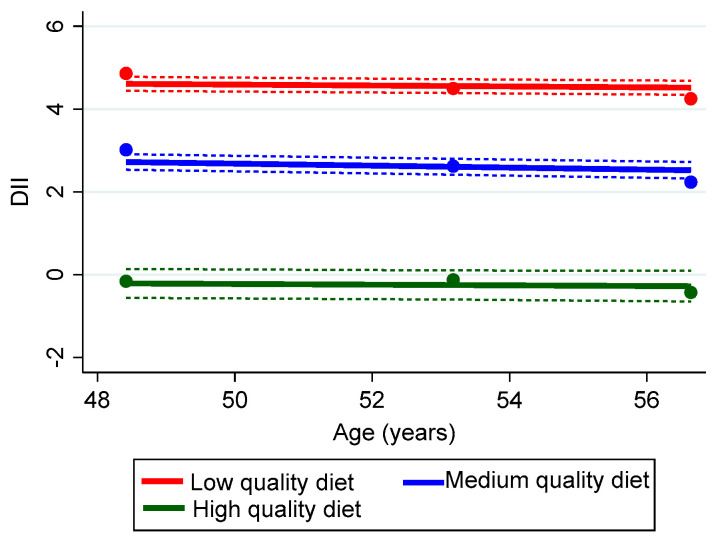
Group Trajectories of Diet Quality using the Dietary Inflammatory Index (DII) for the HANDLS study sample, 2004–2017 (n = 2919). Dots represent observed group means, solid lines represent the estimated trajectories, and dashed lines represent error estimates. Percentage of sample/group trajectory: Low, 32.3%; Medium 57.6%; High, 10.1%.

The findings for the DII revealed that approximately one-third (32.3%) of the HANDLS sample was defined by the pro-inflammatory diet depicted by the red trajectory. The direction of the DII scores is the reverse of the HEI and MAR, with high scores indicating a poor-quality diet. In comparison, an estimated 10.1% of the sample consumed a diet with low-inflammatory potential (high diet quality), as depicted by the green color in Figure 3. Over half the sample (57.6%) had a trajectory indicating middle values of DII, as indicated by the blue trajectory line. Their scores reflected a diet with pro-inflammatory potential (Figure 3). The DII scores revealed declines over visits in all three trajectory groups, which translates into a diet with less inflammatory potential. The mean (SD) Bayesian estimators of the annualized rate of change in quality were −0.09 (0.009) (95% CI [−0.06, −0.10]) for the low, −0.08 (0.007) (95% CI [−0.06, −0.09]) for the middle, and −0.08 (0.011) (95% CI [−0.07, −0.11]) for the high trajectories.

**Figure 4:**
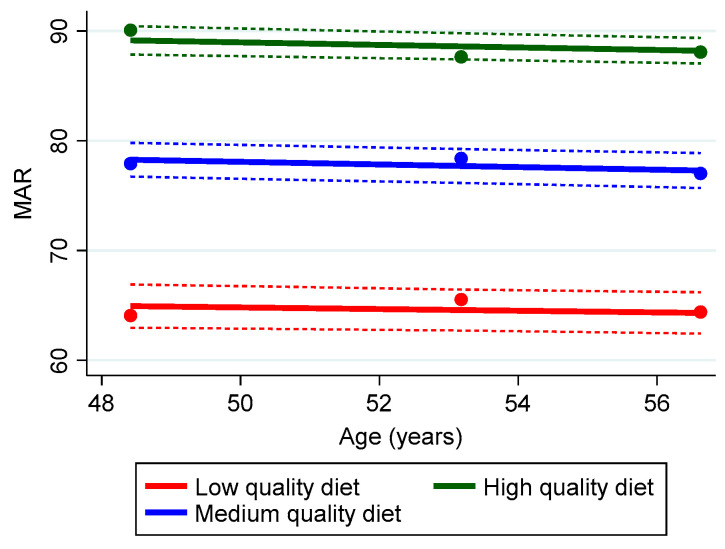
Group Trajectories of Diet Quality using the Mean Nutrient Adequacy (MAR) Score for the HANDLS study sample, 2004–2017 (n = 2919). Dots represent observed group means, solid lines represent the estimated trajectories, and dashed lines represent error estimates. Percentage of sample/group trajectory: Low, 29.9%; Medium, 43.1%; High, 26.9%.

The trajectory for low diet quality assessed by MAR scores was comprised of approximately 29.9% of the sample depicted in Figure 4 as a red line. This trajectory revealed scores < 67% of recommended intakes, suggesting a risk for micronutrient inadequacies. An estimated 43.1% of the sample consumed a diet of higher quality, as depicted by the blue middle trajectory. The third group, comprising 26.9%, had a trajectory of even higher diet quality, represented by the green color (Figure 4). The mean (SD) Bayesian estimators for each group were −0.08 with a SD of 7.3 × 10^−9^ for group 1, 3.8 × 10^−9^ for group 2, and 4.2 × 10^−9^ for group 3, which suggested the slopes do not change and SDs are extremely small and can be considered 0.

Scatterplot matrices were generated to display how each of the diet quality indices at each visit were correlated with each other and with the probability of belonging to the most unhealthy diet quality group (namely, low HEI, low MAR, and high DII) (See Supplementary Figure S4). Overall agreement among diet trajectory groups for the three diet quality indices was as follows: agreement between HEI-2010 and DII was 60% (1759/2919), between MAR and DII, 56% (1647/2919), and between HEI-2010 and MAR, 40% (1171/2919). The weighted kappa (±SE), a measure of agreement in classification, between the HEI-2010 and DII was 0.34 ± 0.01 and between the MAR and DII was 0.40 ± 0.01. These weighted kappa values revealed that the DII had better agreement with the HEI-2010 and MAR indices compared with the agreement between the HEI-2010 and MAR (0.14 ± 0.01).

The results of the mixed-effects regression analyses provide an assessment of the stability of the diet quality trajectories over time, defined as the number of years between visits (Table 3, Table 4 and Table 5). For each quality index, the middle and high diet quality groups were compared with the low diet quality group (the common referent). For the HEI-2010 index, the interaction of time with the middle quality group was significant (β ± SE, 0.156 ± 0.578, *p* = 0.007), indicating that there were significant differences in the rate of diet quality change between this group trajectory and the low diet quality trajectory. For the DII index, a different interaction, time with high diet quality, was found significant (β ± SE, −0.054 ± 0.019, *p* = 0.004). Significant interactions were found for both the middle and high diet quality groups with time for the MAR index. The β (±SE) coefficient for the middle quality interaction with time was −0.390 (0.090) (*p* < 0.001), and for the high quality it was −0.583 (0.099) (*p* < 0.001). Compared with the low diet quality group, the rates of decline varied and could be described as unstable. The full models were adjusted for age at *v* 1, race, sex, and poverty status. 

Table 6 provides the relative odds ratios (OR) associated with the lowest diet quality trajectories compared with those in the other two trajectory groups. For the HEI, the odds of belonging to the middle versus low diet quality group trajectory increased by 1% per year of baseline age and were 38% greater among men versus women, 28% less among African American versus White adults, and 54% greater among individuals living below poverty compared with those living above poverty. Belonging to the high HEI diet quality group in comparison with the low diet quality group’s trajectory was 3% lower with each year of age from baseline, 36% lower among men versus women, 53% lower among African American adults than White adults, and 59% lower among individuals living below poverty compared with those living above poverty.

With respect to the DII and MAR indices, the odds of belonging to either the middle or high diet quality group trajectory versus the low diet quality group were greater for men compared with women, lower among African American adults compared with White adults, and lower among individuals living below poverty compared with those living above poverty (Table 6). 

## 4. Discussion

This study is unique with respect to the diet quality indices chosen to be examined. Each index provided a picture of a different aspect of the quality of the dietary patterns associated with a sample that is underrepresented in the nutrition literature. Yet GBTM yielded three dietary pattern groups for each index, revealing low, medium, and high dietary quality. Based on these trajectories, changes in dietary quality over time were successfully evaluated.

Within each group’s trajectory, there appear to be only small changes in diet quality scores over the past 13 years. The annualized rate of change in diet quality per person was similar given the 95% CI for the Bayesian estimates of the means across the group trajectories within each diet quality index. The observed changes were consistent with the findings of other researchers, despite differences in dietary collection methods. Mertens et al. reported positive 1.83 and 2.81 HEI-2010 scores, based on 3-day records, over 10 years in a Flemish sample of men and women, 18–75 years, respectively [48]. The dietary intakes, collected by the food frequency method, of a sample of Australian women (n = 8161) studied over 12 years were assessed by the Australian Recommended Food Score (ARFS) [49]. The diet quality of 2723 (33%) women improved, defined as an increase ≥4 points (5% of the maximal 74 score) of their original ARFS, while the ARFSs of 29% of women declined by ≤4 points, indicating diet quality worsened. ARFSs of the remaining sample (n = 3077, 38%) were judged stable, defined as a value within ±3 points (4% of the maximal 74 score) of their original score [49]. In contrast to our finding of three diet quality trajectories, Liu et al. found four longitudinal diet quality trajectories for Chinese adults, 20 years and older (n = 6398) over 10 years [50]. Using the Chinese Healthy Eating Index based on 3 days of 24 h recalls, two trajectories showed movement among quality levels, such as the low-moderate-low quality trajectory. One trajectory revealed improved diet quality over time, while another showed worsening quality [50]. The change in DII over time of the HANDLS study sample was less than that of participants in the Multiethnic Cohort Study [30] and of postmenopausal women in the Women’s Health Initiative study [51]. Neither DII study used GBTM.

The mixed-model regression results of this study revealed changes in diet quality over time for all indices. No consistent trend was observed across indices. Even though this change was statistically significant, the actual point value change in scores was small and may not be of practical clinical significance. These small but inconsistent changes are likely associated with volatility in the prices and availability of foods, especially because participants were not systematically retested at the same time of year, making it impossible to adjust for seasonal variations.

The Dietary Patterns Method Project provided researchers with evidence of consistent classifications of adults between pairs of diet quality indices such as the HEI-2010 and Dietary Approach to Stop Hypertension (DASH) [52]. In this study, consistent classifications between dietary indices of similar quality were not always found. This finding was not unexpected since the indices had different scoring methods and shared few common components. Each dietary quality index represented a unique combination of dietary constituents, and only the HEI-2010 was energy adjusted. High HEI-2015 scores translate into more anti-inflammatory, energy-adjusted DII scores, suggesting DII may need to be energy-adjusted when compared with other diet quality indices [53]. 

Even though the HEI and DII scores showed improvement over time, it is evident that participants in the HANDLS study could further improve the quality of their diets by making different food and beverage choices. The groups at higher risk for lower diet quality were those below poverty status, African American adults, and women. Similar to our findings, others reported that non-Hispanic Blacks had lower diet quality (HEI-2015) than non-Hispanic White adults, but they found that women had significantly higher HEI scores than men [54]. These researchers suggested that a negative change in economic circumstances may not account for a worsening in diet quality, with Americans shifting from eating away from home to preparing food at home. In our sample, ~74% of the below-poverty status group, compared with ~65% of the above-poverty status group, reported consuming foods at home. Given the wording of the question included in the AMPM, there is no way to tell if these foods were also prepared at home. The literature also supports our finding that lower-quality diets were associated with groups of lower socioeconomic status. These diets generally cost less per calorie [55,56,57]. 

There is evidence that greater adherence to the *Dietary Guidelines for Americans* is associated with a lower risk of total mortality from cardiovascular disease, cancer, and respiratory diseases in different racial and ethnic groups [58]. In 2011, MyPlate, the US government’s resource to help consumers make healthful food choices consistent with the *Dietary Guidelines for Americans,* was launched. Yet, between 2017 and 2020, only 25.3% of the participants in the National Health and Nutrition Examination Survey reported they were aware of the existence of MyPlate, and fewer (8.3%) used MyPlate to follow dietary recommendations [59]. If more of the public were aware that small improvements in diet quality could impact their health and life expectancy, perhaps they would change their intake from a Western-type pattern to a more optimal pattern, a plant-based pattern abundant in legumes, whole grains, and nuts. Increases in life expectancy would potentially vary with the age at which dietary change was initiated. For instance, improving diet quality at age 60 could increase life expectancy in US women by 8 years and in men by 8.8 years (7). Improvements in diet quality also have the potential to positively affect quality of life [60]. For health professionals, our continual challenge is how to motivate people to initiate exchanging unhealthy food choices for healthy ones and then sustain healthy eating practices given their socioeconomic situation and lifestyle. 

This study has strengths and limitations. First, we utilized longitudinal data over a 10- to 12-year span from a biracial sample underrepresented in the literature to determine the diet quality trajectories. At each visit, 24 h recalls were collected on separate days, resulting in the representation of weekdays and weekend days in the dataset. The AMPM, a validated recall method that reduces measurement error in dietary data collection [61], was used at each visit. Another strength is the use of different indices of diet quality, with consistency in the results between the HEI-2010 and DII. Additionally, the use of GBTM allowed the classification of persons into distinct subgroups whose trajectory membership can be used later to explore their impact on various aspects of health. Lastly, the group trajectories selected from GBTM were confirmed by spaghetti plots. Despite the strengths of the AMPM, self-reported dietary information is subject to social desirability bias and misreporting of food and beverage intake [62]. Given the sampling strategy used for the HANDLS study, the results could be generalized to adults in urban settings with similar characteristics but are not representative of a national sample. Energy adjustments to the DII and MAR scores might result in better comparability with the HEI scores. With respect to GBTM, there are also limitations, such as subjective decision-making about the number of groups, whether to have quadratic terms for all or some of them, and how to determine which combination has the best fit [63].

## 5. Conclusions

In conclusion, three diet quality trajectories, specifically low, middle, and higher quality, were identified for each quality index explored. Mixed-effects regression analyses provided evidence of a significant but minimal change in diet quality over time, regardless of the diet quality index used. These consistencies in the results suggest that a single assessment may be sufficient to characterize typical patterns.

## Figures and Tables

**Table 1:** Descriptive characteristics of HANDLS study sample at baseline: overall, by sex, and by race.

	Overall	Sex			Race		
	n = 2919	Men n = 1261	Women n = 1640	*p*	African American n = 1724	White n = 1177	*p*
Age, *v* 1, X ± SE	48.5 ± 0.2	48.3 ± 0.3	48.5 ± 0.2	0.662	48.3 ± 0.2	48.7 ± 0.3	0.298
Sex, % Men	43.5	-	-		43.2	43.8	0.760
Race, % African American	59.6	59.2	59.8	0.760	-	-	
% below poverty status	41.4	37.7	44.2	<0.001	48.0	31.7	<0.001
Education, %							
<High School	6.6	7.5	5.9	0.161	4.9	9.1	<0.001
High School	60.2	60.9	59.8	base	64.5	53.9	base
>High School	33.2	31.7	34.3	0.235	30.6	37.0	<0.001

**Table 2:** Diet quality indices at each visit by group-based trajectory categories for HANDLS study participants (n = 2919) ^1^.

Index	Low Trajectory			Middle Trajectory			High Trajectory		
	*Visit* 1	*Visit* 2	*Visit* 3	*Visit* 1	*Visit* 2	*Visit* 3	*Visit* 1	*Visit* 2	*Visit* 3
HEI-2010	35.7 ± 0.2	38.2 ± 0.2	40.0 ± 0.3	47.8 ± 0.3	51.3 ± 0.3	54.0 ± 0.3	65.2 ± 1.0	69.3 ± 0.8	71.9 ± 0.7
DII	5.07 ± 0.04	4.71 ± 0.04	4.48 ± 0.05	2.85 ± 0.05	2.49 ± 0.04	2.12 ± 0.04	−0.67 ± 0.13	−0.55 ± 0.12	−0.87 ± 0.11
MAR	59.2 ± 0.7	61.8 ± 0.8	61.0 ± 0.7	77.4 ± 0.3	78.0 ± 0.3	76.4 ± 0.3	91.4 ± 0.3	88.4 ± 0.2	89.01 ± 0.2

^1^ Diet quality indexes were categorized as low, middle, and high, and the estimated mean ± standard error for diet quality indexes are shown. Means adjusted for age, sex, race, and income. Abbreviations: HEI—Healthy Eating Index; DII—Dietary Inflammatory Index; MAR—Mean Adequacy Ratio.

**Table 3:** Adjusted mixed-effects linear regression models between HEI-2010 diet quality trajectory groups with time-dependent HEI-2010 diet quality index.

HEI-2010	Coefficient	SE	t	*p*
Time	0.617	0.066	9.38	<0.001
Trajectory Group 2	12.416	0.330	37.68	<0.001
Trajectory Group 3	30.651	0.757	40.48	<0.001
Time × Trajectory Group 2	0.156	0.578	2.70	0.007
Time × Trajectory Group 3	0.110	0.124	0.89	0.375
Age, visit 1, centered	0.244	0.172	14.17	<0.001
Time × Age	−0.003	0.003	−0.97	0.330
Sex, Men	−0.101	0.323	−0.31	0.756
Time × Sex, Men	−0.071	0.057	−1.26	0.209
Race, African American (AA)	0.755	0.329	2.29	0.022
Time × Race (AA)	−0.140	0.059	−2.37	0.018
Below poverty status, <125%	−0.848	0.332	−2.55	0.011
Time × Below poverty status	0.006	0.058	0.11	0.916
Cons	36.014	0.354	101.64	<0.001

Comparison with low diet quality trajectory as a common referent category. Group 2—Middle quality. Group 3—High quality.

**Table 4:** Adjusted mixed-effects linear regression models between DII diet quality trajectory groups with time-dependent DII diet quality index.

DII	Coefficient	SE	t	*p*
Time	0.091	0.012	7.31	<0.001
Trajectory Group 2	2.241	0.061	36.59	<0.001
Trajectory Group 3	5.624	0.108	51.98	<0.001
Time × Trajectory Group 2	0.002	0.011	0.21	0.831
Time × Trajectory Group 3	−0.054	0.019	−2.88	0.004
Age, visit 1, centered	0.020	0.003	6.74	<0.001
Time × Age	−0.001	0.0005	−2.04	0.041
Sex, Men	0.232	0.056	4.13	<0.001
Time × Sex, Men	−0.006	0.010	−0.62	0.534
Race, African American (AA)	0.189	0.057	3.28	0.001
Time × Race (AA)	0.007	0.010	0.63	0.529
Below poverty status, <125%	0.107	0.058	1.85	0.064
Time × Below poverty status	−0.042	0.010	−4.23	<0.001
Cons	−5.071	0.067	−75.73	<0.001

Comparison with low diet quality trajectory as a common referent category. Group 2—Middle quality. Group 3—High quality.

**Table 5:** Adjusted mixed-effects linear regression models: associations of MAR diet quality trajectory groups within time-dependent MAR diet quality index.

MAR	Coefficient	SE	t	*p*
Time	0.298	0.096	3.12	0.002
Trajectory Group 2	18.221	0.507	35.96	<0.001
Trajectory Group 3	31.352	0.564	55.64	<0.001
Time × Trajectory Group 2	−0.390	0.090	−4.36	<0.001
Time × Trajectory Group 3	−0.583	0.099	−5.87	<0.001
Age, visit 1, centered	0.001	0.022	0.03	0.977
Time X Age	−0.025	0.004	−6.42	<0.001
Sex, Men	0.524	0.411	1.28	0.202
Time × Sex, Men	0.040	0.072	0.56	0.577
Race, African American (AA)	0.205	0.416	0.49	0.621
Time × Race (AA)	−0.018	0.075	−0.24	0.810
Below poverty status, <125%	0.683	0.415	1.65	0.100
Time × Below poverty status	−0.182	0.072	−2.53	0.011
Cons	58.941	0.520	113.44	<0.001

Comparison with low diet quality trajectory as a common referent category. Group 2—Middle quality. Group 3—High quality.

**Table 6:** Relative Odds Ratios (OR) for group membership in middle and high diet quality trajectory groups compared with the common referent of low diet quality trajectories groups for each of 3 diet quality indices.

	Healthy Eating Index-2010 (HEI)				Diet Inflammatory Index (DII)				Mean Adequacy Ratio (MAR)			
*Covariates* ^1^	*Middle*		*High*		*Middle*		*High*		*Middle*		*High*	
	OR ± SE	*p*	OR ± SE	*p*	OR ± SE	*p*	OR ± SE	*p*	OR ± SE	*p*	OR ± SE	*p*
Age, Visit 1	1.01 ± 0.4 × 10^−2^	0.001	0.97 ± 0.01	0.007	0.97 ± 0.4 × 10^−2^	<0.001	0.95 ± 0.01	<0.001	1.00 ± 0.01	0.288	1.00 ± 0.01	0.523
Sex	1.38 ± 0.11	<0.001	0.64 ± 0.12	0.018	1.65 ± 0.14	<0.001	2.10 ± 0.32	<0.001	1.85 ± 0.19	<0.001	2.66 ± 0.29	<0.001
Race	0.72 ± 0.06	<0.001	0.47 ± 0.09	<0.001	0.90 ± 0.08	0.198	0.40 ± 0.06	<0.001	0.93 ± 0.09	0.483	0.60 ± 0.07	<0.001
Poverty status	1.54 ± 0.12	<0.001	0.41 ± 0.09	<0.001	0.69 ± 0.06	<0.001	0.41 ± 0.07	<0.001	0.80 ± 0.08	0.020	0.68 ± 0.07	<0.001

^1^ Reference group—Sex: men, Race: AA, Poverty status: <125% Federal poverty guidelines, SE: Standard Errors for Ln (OR).

## Data Availability

Data are available upon request to researchers with valid proposals who agree to the confidentiality agreement as required by our Institutional Review Board. We publicize our policies on our website https://handls.nih.gov, accessed on 6 July 2023. Requests for data access may be sent to Alan Zonderman (co-author) or the study manager, Jennifer Norbeck, at norbeckje@mail.nih.gov.

## Supplementary Materials

The following supporting information can be downloaded at: https://www.mdpi.com/article/10.3390/nu15143099/s1, Figure S1: Spaghetti plot of HEI diet quality group trajectories; Figure S2: Spaghetti plot of DII diet quality group trajectories; Figure S3: Spaghetti plot of MAR diet quality group trajectories; Figure S4: Scatterplots displaying correlation of diet quality scores between visits by index for the HANDLS study sample; Table S1: Group trajectory membership by diet quality index title.
