# Supplementary material for: Diet Quality Trajectories over Adulthood in a Biracial Urban Sample from the Healthy Aging in Neighborhoods of Diversity across the Life Span Study
Source: Nutrients. 2023 Jul 11;15(14):3099. doi: 10.3390/nu15143099 (PMC10383268; doi:10.3390/nu15143099)
Supplement: Supplementary file 1 [file nutrients-15-03099-s001.zip › nutrients-2467380-supplementary.pdf]

Supplementary analyses

Figure S1. Spaghetti plot of HEI diet quality group trajectories

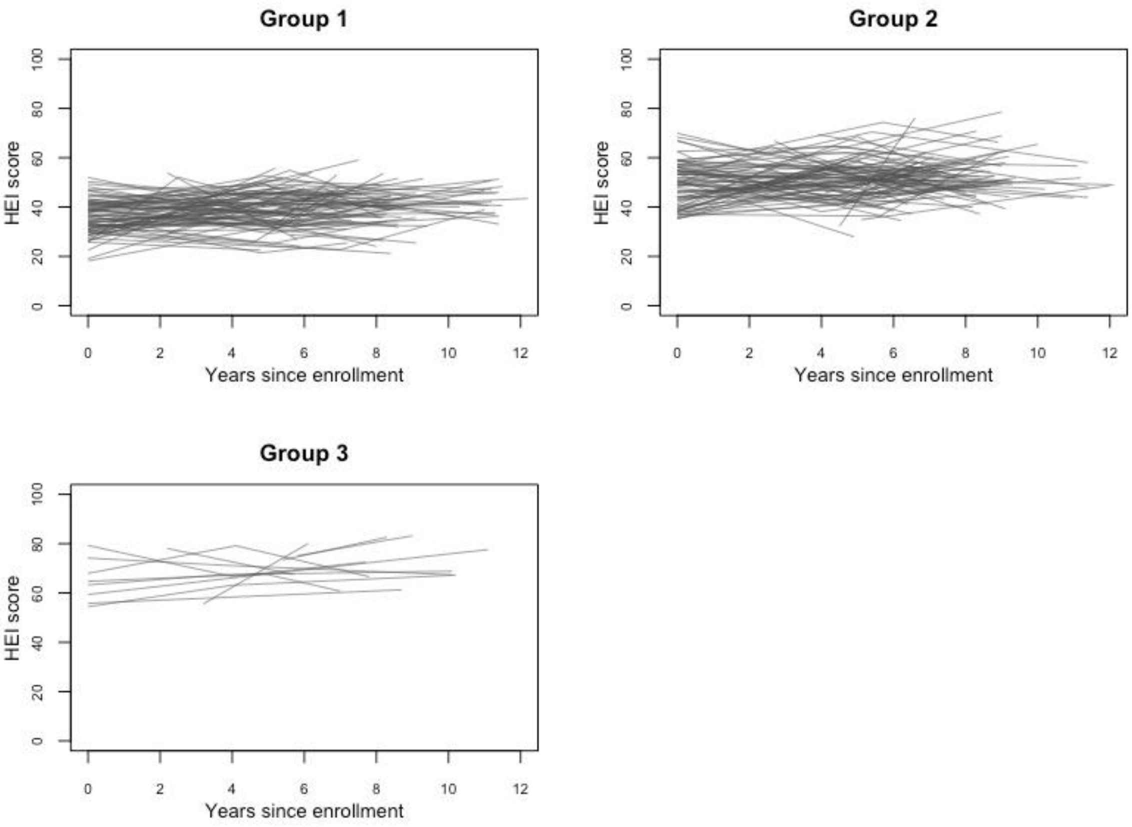

Figure S2. Spaghetti plot of DII diet quality group trajectories

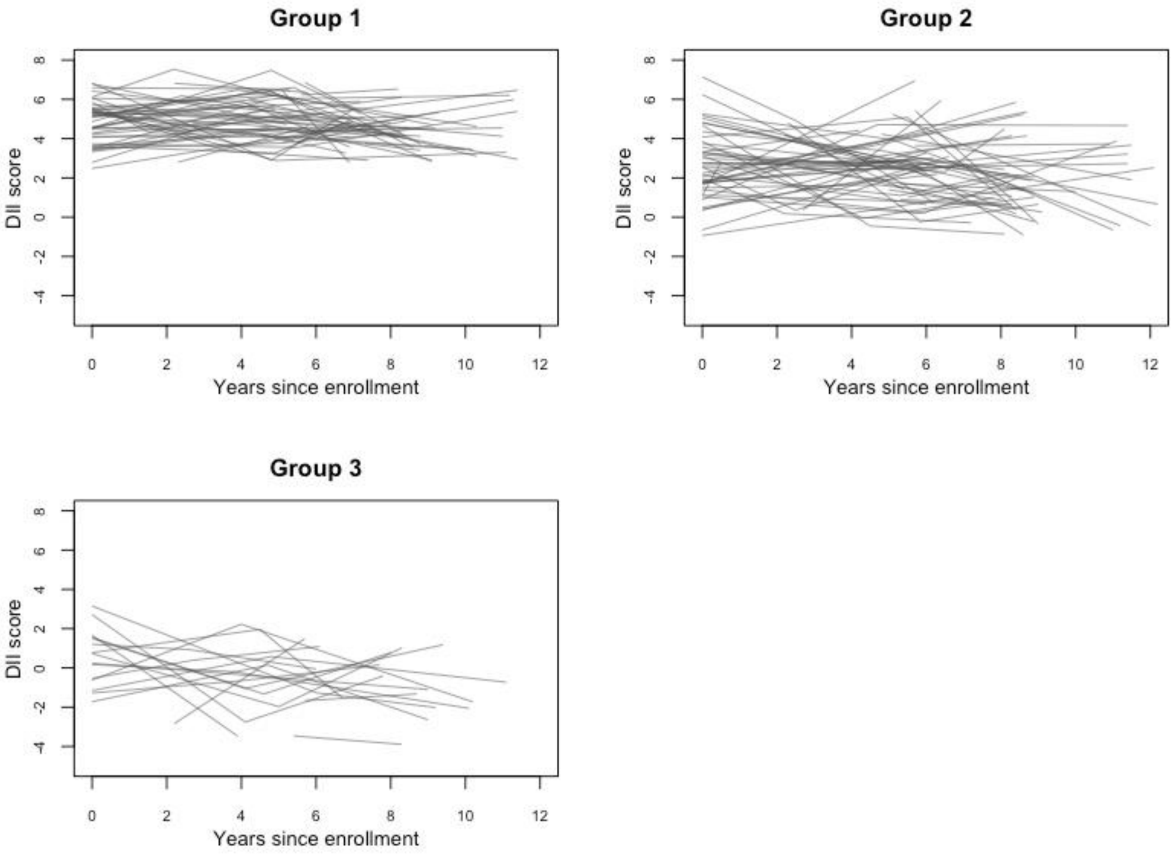

Figure S3. Spaghetti plot of MAR diet quality group trajectories

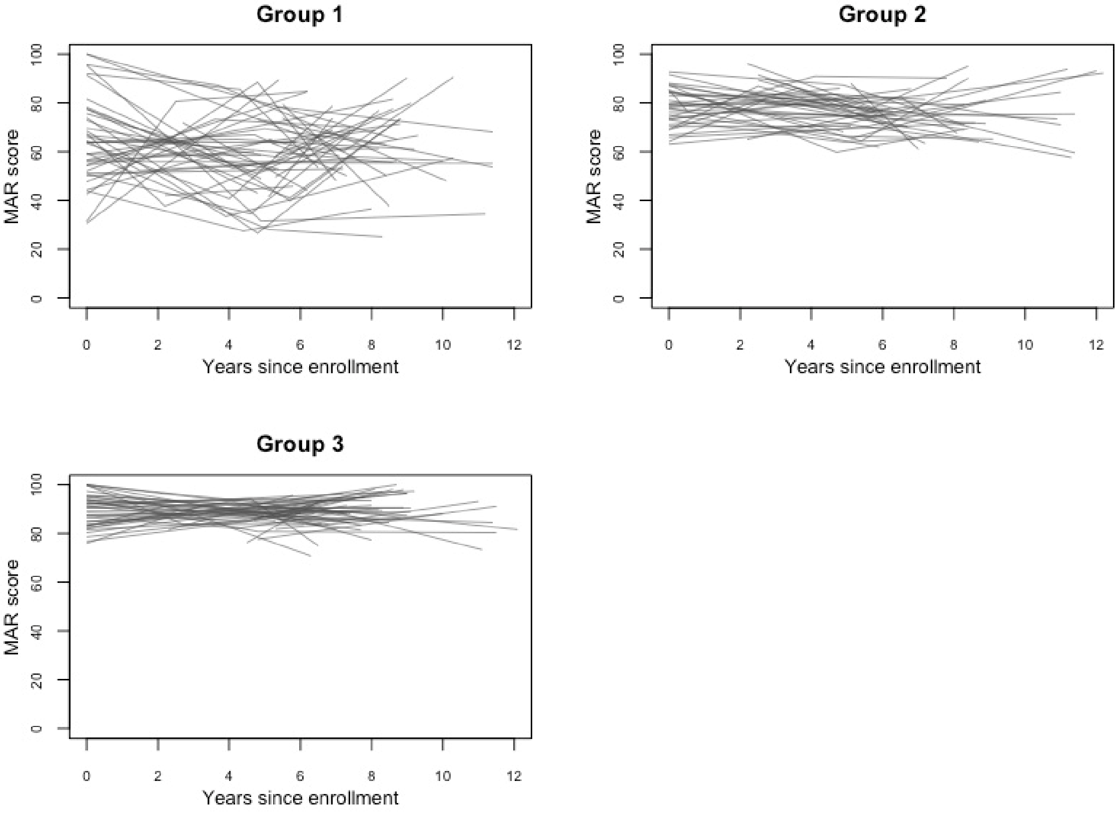

Figure S4. Scatterplots displaying correlation of diet quality scores between visits by index for HANDLS study sample

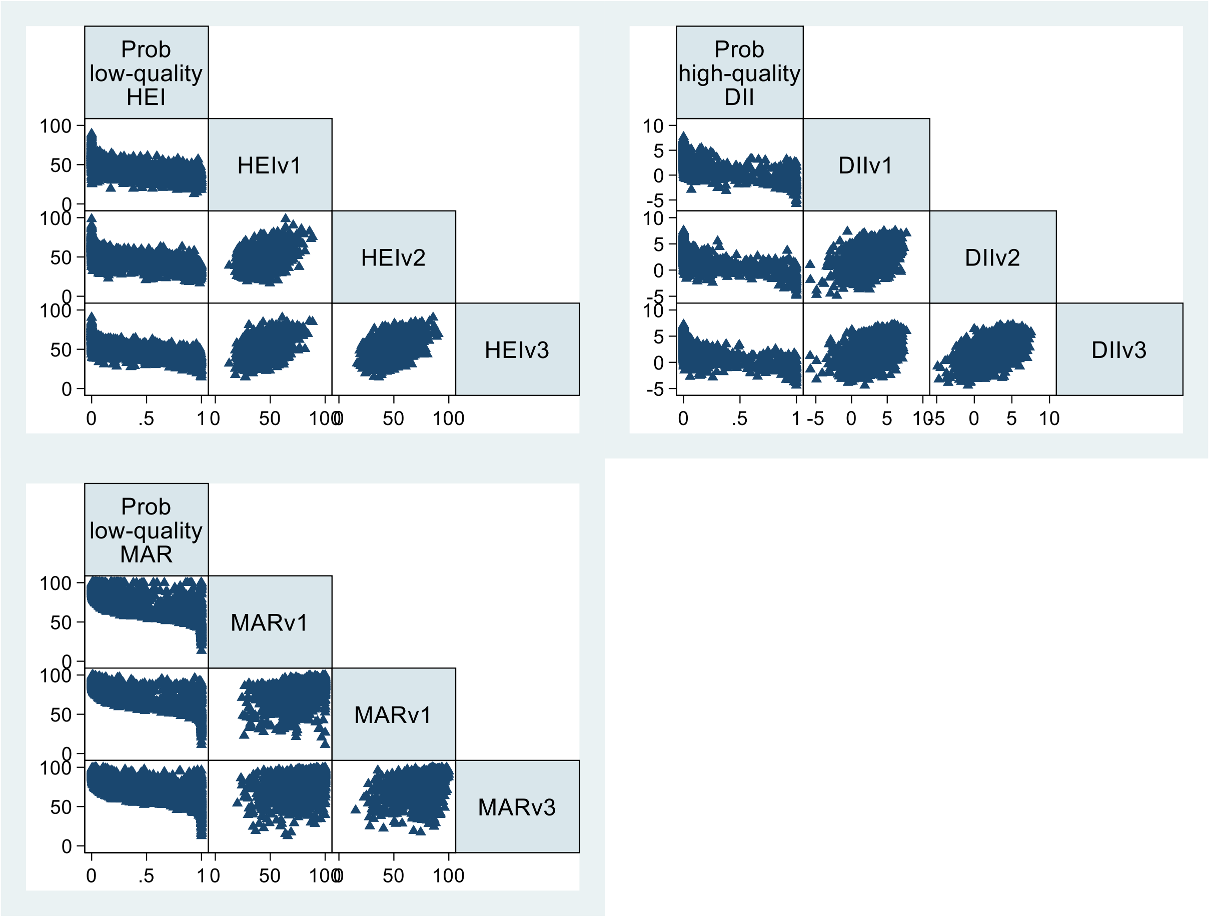

Abbreviations: HEI-Healthy Eating Index-2010, DII – Dietary Inflammatory Index, MAR- Mean Nutrient Adequacy, v-visit, y-axis displays diet quality index score with the range relevant to the index, x-axis, correlation statistic ranging from 0 to 1.

Table S1. Group trajectory membership by diet quality index

Healthy Eating Index

Group	Parameter	Estimate	Standard Error	T for H0: Parameter=0	Prob > T
1	Intercept	29.87102	1.29335	23.096	0
	Linear	0.37483	0.02779	13.49	0
2	Intercept	31.14183	1.21663	25.597	0
	Linear	0.14664	0.02393	6.127	0
3	Intercept	50.03678	3.37714	14.816	0
	Linear	0.33477	0.06189	5.409	0
1	Sigma	9.85871	0.18013	54.731	0
2	Sigma	8.26014	0.22219	37.176	0
3	Sigma	9.54805	0.60493	15.784	0

Group membership

1	(%)	48.29078	4.18941	11.527	0.0000
2	(%)	46.07834	4.76126	9.678	0.0000
3	(%)	5.63088	1.09093	5.162	0.0000

BIC=-24380.79 (N=6383) BIC=-24376.49 (N=2919) AIC=-24343.61 ll= -24332.61

Parameter estimates for adding risk factors

29.87102, 0.37483, 31.14183, 0.14664, 50.03678, 0.33477,
9.85871, 8.26014, 9.54805, -0.04690, -2.14898

Parameter estimates

29.87102, 0.37483, 31.14183, 0.14664, 50.03678, 0.33477,
9.85871, 8.26014, 9.54805, 48.29078, 46.07834, 5.63088

Entropy = 0.579

Dietary Inflammatory Potential

Group	Parameter	Estimate	Standard Error	T for H0: Parameter=0	Prob > T
1	Intercept	3.8932	0.20858	18.666	0
	Linear	-0.02419	0.0039	-6.206	0
2	Intercept	5.17053	0.22325	23.16	0
	Linear	-0.01158	0.00393	-2.944	0.0033
3	Intercept	0.17847	0.54678	0.326	0.7441
	Linear	-0.00806	0.01028	-0.784	0.4331
1	Sigma	1.69323	0.02734	61.928	0
2	Sigma	1.25856	0.04221	29.815	0
3	Sigma	1.82018	0.07199	25.285	0

Group membership

1	(%)	57.65107	2.85709	20.178	0.0000
2	(%)	32.25760	3.34801	9.635	0.0000
3	(%)	10.09134	1.42685	7.072	0.0000

BIC=-13285.80 (N=6383) BIC=-13281.50 (N=2918) AIC=-13248.62 ll= -13237.62

Parameter estimates for adding risk factors

3.89320, -0.02419, 5.17053, -0.01158, 0.17847, -0.00806,
1.69323, 1.25856, 1.82018, -0.58066, -1.74273

Parameter estimates

3.89320, -0.02419, 5.17053, -0.01158, 0.17847, -0.00806,
1.69323, 1.25856, 1.82018, 57.65107, 32.25760, 10.09134

Entropy = 0.594

Mean Adequacy Ratio

Group	Parameter	Standard Estimate	T Error	for	HO: Parameter	Prob>T
1	Intercept	68.82505	2.67874		25.693	0
	Linear	-0.07735	0.04741		-1.631	0.1029
2	Intercept	95.19269	1.48648		64.039	0
	Linear	-0.12109	0.02519		-4.807	0
3	Intercept	84.1768	1.61125		52.243	0
	Linear	-0.12099	0.02991		-4.046	0.0001
1	Sigma	17.15801	0.39113		43.868	0
2	Sigma	6.87085	0.24368		28.196	0
3	Sigma	9.71419	0.34143		28.452	0

Group membership

1	(%)	29.93478	2.67402	11.195	0.0000
2	(%)	26.94840	2.75761	9.772	0.0000
3	(%)	43.11682	2.67681	16.108	0.0000

BIC=-25218.99 (N=6383) BIC=-25214.68 (N=2919) AIC=-25181.80 ll= -25170.80

Parameter estimates for adding risk factors

68.82505, -0.07735, 95.19269, -0.12109, 84.17680, -0.12099,
17.15801, 6.87085, 9.71419, -0.10510, 0.36489

Parameter estimates

68.82505, -0.07735, 95.19269, -0.12109, 84.17680, -0.12099,
17.15801, 6.87085, 9.71419, 29.93478, 26.94840, 43.11682

Entropy = 0.485
